# Experience of Low-Pass Whole-Genome Sequencing-Based Copy Number Variant Analysis: A Survey of Chinese Tertiary Hospitals

**DOI:** 10.3390/diagnostics12051098

**Published:** 2022-04-27

**Authors:** Yu Zheng, Baosheng Zhu, Jichun Tan, Yichun Guan, The Chinese Genomic Structural Variants Consortium, Cynthia C. Morton, Guangxiu Lu

**Affiliations:** 1 Prenatal Genetic Diagnosis Centre, Department of Obstetrics & Gynecology, Prince of Wales Hospital, The Chinese University of Hong Kong, Hong Kong, China; haleyzheng@link.cuhk.edu.hk; 2 Department of Genetics Medicine, First People’s Hospital of Yunnan Province, Kunming 650021, China; bszhu@aliyun.com; 3 Reproductive Medicine Centre, Shengjing Hospital of China Medical University, Shenyang 110055, China; tjczjh@163.com; 4 Reproductive Medicine Centre, Third Affiliated Hospital of Zhengzhou University, Zhengzhou 450052, China; lisamayguan@126.com; 5 Department of Obstetrics and Gynecology and Pathology, Brigham and Women’s Hospital and Harvard Medical School, Boston, MA 02115, USA; 6 Broad Institute of MIT and Harvard, Cambridge, MA 02142, USA; 7 Manchester Centre for Audiology and Deafness (ManCAD), School of Health Sciences, University of Manchester, Manchester M13 9PL, UK; 8 Genetics Centre, Reproductive & Genetic Hospital of CITIC-Xiangya, Changsha 410008, China

**Keywords:** low-pass whole-genome sequencing, copy number variant, tertiary hospital, laboratory experience, variant nomenclature

## Abstract

In China, low-pass whole-genome sequencing (low-pass WGS) is emerging as an alternative diagnostic test to detect copy number variants (CNVs). This survey aimed to study the laboratory practice, service quality, and case volumes of low-pass WGS-based CNV analysis among national accredited Chinese tertiary hospitals that have routinely applied low-pass WGS for more than a year and that have been certified in next-generation sequencing (NGS) clinical applications for more than three years. The questionnaire focused on (1) the composition of patients’ referral indications for testing and annual case volumes; (2) the capacity of conducting laboratory assays, bioinformatic analyses, and reporting; (3) the sequencing platforms and parameters utilized; and (4) CNV nomenclature in reports. Participants were required to respond based on their routine laboratory practices and data audited in a 12-month period from February 2019 to January 2020. Overall, 24 participants representing 24 tertiary referral hospitals from 21 provincial administrative regions in China returned the questionnaires. Excluding three hospitals routinely applying low-pass WGS for non-invasive prenatal testing (NIPT) only, the analysis only focused on the data submitted by the rest 21 hospitals. These hospitals applied low-pass WGS-based CNV analysis for four primary applications: high-risk pregnancies, spontaneous abortions, couples with adverse pregnancy history, and children with congenital birth defects. The overall estimated annual sample volume was over 36,000 cases. The survey results showed that the most commonly reported detection limit for CNV size (resolution) was 100 kb; however, the sequencing methods utilized by the participants were variable (single-end: 61.90%, 13/21; paired-end: 28.57%, 6/21; both: 9.52%, 2/21). The diversity was also reflected in the sequencing parameters: the mean read count was 13.75 million reads/case (95% CI, 9.91–17.60) and the read-length median was 65 bp (95% CI, 75.17–104.83). To assess further the compliance of the CNV reporting nomenclature according to the 2016 edition of International System for Human Cytogenomics Nomenclature (ISCN 2016), a scoring metric was applied and yielded responses from 19 hospitals; the mean compliance score was 7.79 out of 10 points (95% CI, 6.78–8.80). Our results indicated that the low-pass WGS-based CNV analysis service is in great demand in China. From a quality control perspective, challenges remain regarding the establishment of standard criteria for low-pass WGS-based CNV analysis and data reporting formats. In summary, the low-pass WGS-based method is becoming a common diagnostic approach, transforming the possibilities for genetic diagnoses for patients in China.

## 1. Introduction

Copy number variants (CNVs) are defined as DNA segments of at least 50 base-pair (bp) in size that present at a variable copy number compared with a representative reference genome [1]. CNVs contribute to genome diversity in the human population [2] and are also associated with an abundance of human disorders [3], such as DiGeorge syndrome (OMIM# 188400) caused by a 1.5- to 3.0-Mb heterozygous deletion of chromosome 22 in band q11.2, and Charcot–Marie–Tooth disease, type 1A (OMIM# 118220) due to duplication or mutation in the gene-encoding peripheral myelin protein-22 (*PMP22*; 601097) in 17p12. In the past decade, chromosomal microarray analysis (CMA) has been adopted as a first-tier test for the detection of clinically significant CNVs [4,5]. However, CMA is limited by its relatively low throughput and uneven probe coverage. Probes are not evenly distributed across the genome and may miss some clinically significant CNVs [6,7]. In recent years, low-pass whole-genome sequencing (low-pass WGS) began to be utilized for CNV detection with the development of different next-generation sequencing (NGS) platforms as well as numerous computational analytical tools [8,9]. Compared with routine CMA, low-pass WGS provides better genome-wide coverage (reads much more evenly aligned to most of the genomic regions) resulting in higher resolution of genome-wide CNV detection and higher sensitivity in the detection of low-level mosaicism. In addition, it provides higher throughput and feasibility of automation, a smaller amount of DNA is required, and it results in lower experimental failure rates [10]. Moreover, it shares the same sequencing platform with other NGS-based genetic tests (e.g., non-invasive prenatal testing, NIPT). Compared to high read-depth WGS (usually defined as ~30-fold), which detects single-nucleotide variants (SNVs), small insertions and deletions (indels), CNVs, and structural variations (SVs) simultaneously, low-pass WGS (usually under 1-fold) focuses on CNV analysis (50–100 kb) only. Low-pass WGS requires less manpower for both wet-lab and data-analysis procedures, but has higher throughput and a shorter turnaround time and thus is suitable for laboratories with increasing demands for prenatal diagnosis. In 2019, a joint Chinese expert group consensus was published recommending low-pass WGS as the first tier of diagnostic testing for pregnant women who had been referred for or elected prenatal diagnosis, couples with balanced chromosomal translocations, and/or couples with pregnancy loss [11]. Thereupon, low-pass WGS-based CNV analysis began to be utilized in Chinese laboratories [12,13,14]. However, there were no detailed recommendations about the parameters of the laboratory wet-lab and dry-lab procedures as well as the variant nomenclature guidelines for the low-pass WGS-based approach in China or western countries, leading to difficulties in achieving a consensus and standard practice.

Low-pass WGS-based CNV testing involves three major processes: wet-lab assays (e.g., DNA extraction, library preparation, sequencing), bioinformatic analysis (e.g., alignment, normalization, variant calling, and variant annotation), and reporting (e.g., manual variant review, interpretation) [15,16]. Each step is technically challenging and requires quality control (QC) measures to ensure the reliability of the clinical results [15]. Nowadays, there are various sequencing platforms utilizing different sequencing and analytical parameters, which warrants a comprehensive survey to assess the service quality among providers [9]. According to several studies, the quality of a low-pass WGS-based CNV analysis would be more reliable if a higher sequencing read depth (equivalent to the total read counts multiplied by the length of the reads, then divided by the size of the reference human genome) could be obtained, resulting in less variation of genomic coverage and higher resolution of CNV detection. In addition, longer read lengths and paired-end sequencing (compared to single-end sequencing) also provide more reliable information about the coordinates of the CNV boundaries, thus improving CNV calling [7]. Therefore, primary technical considerations for conducting low-pass WGS-based CNV analysis should include the sequencing platform used, the minimal read amounts, the read length, and the sequencing mode (paired-end or single-end). In general, achieving higher resolutions requires larger amounts of sequencing reads. In terms of the variant nomenclature described in a clinical report, a Chinese expert group published a consensus in 2018 and recommended following the guidelines set by the Human Genome Variation Society (HGVS) and the American College of Medical Genetics and Genomics (ACMG) standards in a clinical NGS service [17]. In 2020, a joint consensus [18] for the clinical interpretation and reporting of CNVs has been published by the ACMG and the Clinical Genome Resource (ClinGen). It is recommended to follow the International System for Human Cytogenomics Nomenclature (ISCN) to describe and report the numerical and/or chromosomal structural changes and the HGVS in describing the single nucleotide-level changes. However, how well the guidelines were compliant in actual practice was unclear.

## 2. Materials and Methods

In March 2020, we designed an online questionnaire (https://www.wenjuan.com/s/nY3MFf2/, accessed on 31 March 2020) and aimed to study the current practices of accredited laboratories, service quality, and case volumes of low-pass WGS-based CNV analysis among Chinese tertiary hospitals. Tertiary hospitals in China refer to a general hospitals that usually have a full complement of services and a higher level of specialized care such as prenatal diagnostic services. It is usually a university-affiliated hospital or women and children’s specialist center, which are equipped with certified prenatal diagnostic laboratories. The inclusion criteria were: Chinese tertiary hospitals that have routinely applied low-pass WGS for more than a year and have been certificated in NGS-based genetic test services for more than three years. For each hospital, one coordinator was assigned as a key informant and was responsible for returning the information from the questionnaires. Participants were required to respond based on their routine diagnostic laboratory practices and data audited in a 12-month period from February 2019 to January 2020. All participants took this opportunity to form the Chinese Genomic Structural Variants Consortium, a working group focusing on improving the quality of service of genome-sequencing-based structural variants detection (including CNVs). In this study, we focused on low-pass WGS for CNVs analysis and did not include the NIPT application. Therefore, hospitals that only applied low-pass WGS for NIPT were excluded from this study.

The background characteristics of the departments of the participants as well as the services provided by their departments were investigated in the survey questionnaire. Based on the routine practice of their departments, the composition of patients’ referrals and annual sample volumes of low-pass WGS-based CNV analysis from February 2019 to January 2020 were assessed. Based on the low-pass WGS-based CNV analysis testing provided by the hospitals, participants were required to provide information on the capacity of their hospitals in conducting (1) wet laboratory assays, (2) bioinformatics analyses, and (3) reporting internally; and the sequencing parameters (platforms, mode, read counts, read length) used. In addition, submissions of a sample report of two representative pathogenic CNVs (DiGeorge Syndrome (DGS) and Charcot–Marie–Tooth Disease type 1A (CMT1A)) were requested to assess their CNV nomenclature compliance with the internationally recognized criteria: ISCN 2016 [19] for chromosome aberrations and the Human Genome Variation Society (HGVS) recommendations (http://varnomen.hgvs.org/, accessed on 31 March 2020) for nucleotide variant nomenclature. The type of the variant, band region, genomic coordinates as well as the genome build of these two CNVs were given: DGS heterozygous deletion: 22q11.2; 22:19009792-21452445 (GRCh37), and CMT1 duplication: 17p12; 17:14097915-15470903 (GRCh37).

Survey data were collected through online questionnaire submissions and further confirmed through phone interviews as well as e-mails for a written record.

## 3. Results

### 3.1. Characteristics of Participants

Overall, 24 Chinese tertiary hospitals (Table 1) responded to this survey, involving 21 out of a total of 34 provincial administrative regions in China. Low-pass WGS-related services provided by the participants could be classified to support general molecular genetic testing (15/24, 62.5%), assisted reproduction (12/24, 50.0%), prenatal diagnosis (9/24, 37.5%), and pediatrics (1/24, 4.2%). The percentage of hospitals that provided low-pass WGS-based CNV analysis testing for diagnostic purpose was 87.5% (21/24) as the other three were routinely applying low-pass WGS for NIPT only. By excluding NIPT, the analysis in the present study only focused on data submitted by 21 hospitals.

### 3.2. Compositions of Patients’ Referrals and Annual Sample Volumes of Low-Pass WGS-Based CNV Analysis

**Figure 1:**
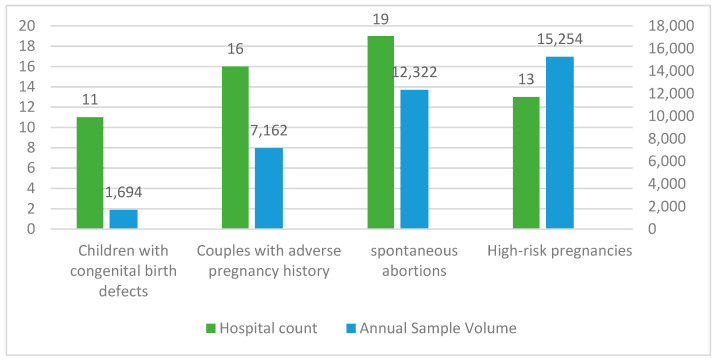
Compositions of patients’ referrals and annual sample volumes of low-pass WGS-based CNV analysis of 21 Chinese tertiary hospitals enrolled in this survey. The green bars illustrate the number of hospitals that provided low-pass WGS-based CNV analysis for each type of referral, which corresponds to the left axis; the blue bars illustrate the annual sample volumes of each type of referral, which corresponds to the right axis.

Low-pass WGS-based CNV analysis was used as a diagnostic tool for patients with the following four indications in these 21 hospitals: spontaneous abortions (90.5%, 19/21), couples with adverse pregnancy history (76.2%, 16/21), high-risk pregnancies (61.9%, 13/21), or children with congenital birth defects (52.4%, 11/21). In total, there were 36,432 cases subjected to annual testing, of whom 41.87% (*n* = 15,254) were high-risk pregnancies, 33.82% (*n* = 12,322) were spontaneous abortions, 19.66% (*n* = 7162) were couples with an adverse pregnancy history, and 4.65% (*n* = 1694) were children with congenital birth defects and suspected genetic disorders, respectively (Figure 1).

### 3.3. The Hospital’s Capacity to Conduct Low-Pass WGS-Based CNV Analysis Internally

**Figure 2:**
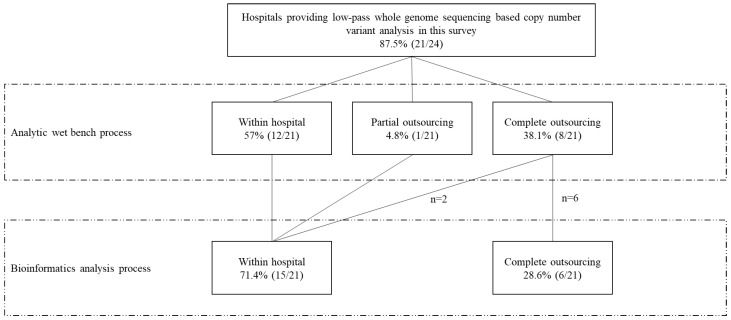
Capacity of conducting low-pass whole-genome sequencing-based copy number variant analysis within hospitals among 21 Chinese tertiary hospitals in this survey.

As shown in Figure 2, 57.1% (12/21) of the hospitals conducted the wet lab work in-house, but 38.1% (8/21) outsourced the wet lab of low-pass WGS. One participant (Hospital 4) conducted the wet lab internally only for the prenatal samples but outsourced the wet lab for the postnatal samples. As for the subsequent bioinformatics analysis process, 71.4% (15/21) of the hospitals analyzed the sequencing data internally, whereas 28.57% (6/21) outsourced this task. Of note, all hospitals conducting the wet-bench process internally also performed the subsequent bioinformatics analysis internally. In contrast, for those outsourcing the wet-bench process, only 25% (2/8) performed the bioinformatics analysis process within their hospital.

### 3.4. Sequencing Platforms and Parameters Utilized for Low-Pass WGS-Based CNV Analysis

Among the 21 surveyed hospitals, sequencing platforms from three manufacturers were used, including MGI Tech Co., Ltd. (MGI) (Shenzhen, China), Illumina (San Diego, CA, USA), and Thermo Fisher Scientific (Waltham, MA, USA). The platforms utilized are shown in Table 2. The majority of the participating hospitals only used one platform except for Hospital 4, which conducted wet lab work internally only for the prenatal samples with approximately 0.04-fold read depth (~3 million read pairs with single-end 38 bp in size) on the NextSeq 500 platform but outsourced the wet lab for the postnatal samples with approximately 5.63-fold read depth (~60 million read pairs with paired-end 150 bp in size) on the HiSeq 2000 platform.

### 3.5. Sequencing Parameters Utilized for Low-Pass WGS-Based CNV Analysis

**Figure 3:**
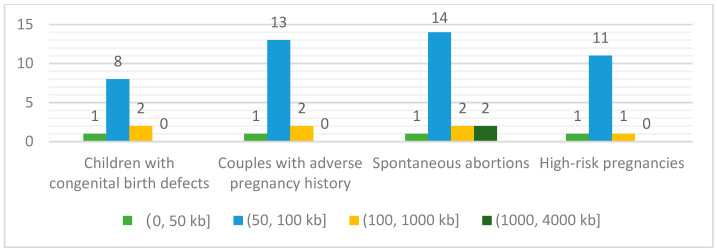
Detection limit for CNV size detection (resolution) of low-pass WGS-based CNV analysis for different patients among 21 Chinese tertiary hospitals in this survey. The green, blue, orange, and dark-green bars show the number of hospitals reporting their resolution within the (0, 50 kb], (50, 100 kb], (100, 1000 kb], (1000, 4000 kb] range across different indications of referral.

**Figure 4:**
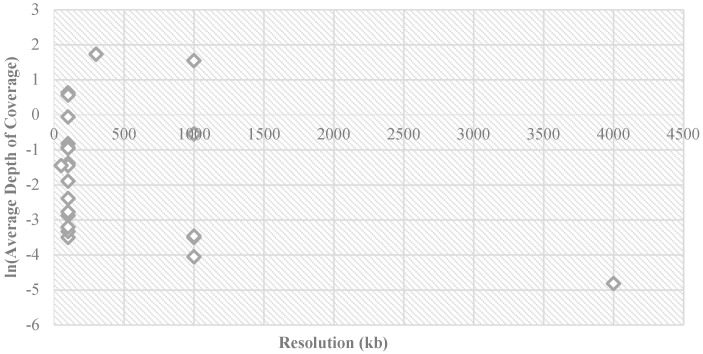
Sequencing parameters used by 21 Chinese tertiary hospitals in this survey. The average depth of coverage (ln value) varied across different reported detection limits for CNV size detection (resolution). Taking the resolution of 100 kb as an example, the average depth of coverage ranged from 0.03 (ln −3.5 in the figure) to 1.88 (ln 0.6 in the figure).

The most frequently reported detection limit of CNV length (resolution) for all primary diagnostic purposes (Figure 3) was 100 kb. The detection limit reported for spontaneous abortion cases ranged from 50 kb to 4 Mb among these 21 hospitals, whereas for the other three applications, the detection limits were within 50 kb to 1 Mb. The hospitals also used different sequencing modes: 61.90% (13/21) of the hospitals used single-end sequencing and 28.57% (6/21) used paired-end. However, Hospital 4 used single-end for the prenatal samples but paired-end for the postnatal samples, whereas Hospital 16 provided both methods for all patients (using paired-end when detection of complex structural variants and absence of heterozygosity was needed). Sequencing parameters also varied: median read-length was 65 bp (95% CI, 75.17–104.83) and the mean read counts was 13.75 million reads/case (95% CI, 9.91–17.60). The average depth of coverage (showing as ln value in Figure 4) also varied with different reported detection limits for the CNV size detection (resolution). Taking the resolution of 100 kb as an example, the average depth of coverage obtained ranged from 0.03 (ln−3.5 in Figure 4) to 1.88 (ln0.6 in Figure 4).

### 3.6. The Compliance of CNV Reporting Nomenclature with Internationally-Recognized Criteria

In total, 19 participants submitted the reporting nomenclatures for DGS (deletion type of CNV) and CMT1A (duplication type of CNV) based on their routine practice with low-pass WGS-based CNV analysis (Table 3). Twelve types of nomenclature were received for both DGS and CMT1A, and the nomenclature of DGS and CMT1A submitted by the same participant followed the same reporting format. The scoring criteria were designed to verify whether each submitted nomenclature was compliant with the internationally recognized criteria: 5 points were awarded for following the ISCN 2016 nomenclature format for chromosomal aberrations; 5 points were awarded for following the HGVS nomenclature format for nucleotide variants; and a deduction of 1 point was made if (1) the symbol of sequencing-based technology was not mentioned in the string; (2) the genome build was not included; (3) the prefix reference sequence “g.” was not present; (4) the affected chromosome was not listed for the ISCN-like or HGVS-like portions; (5) the affected chromosome arm and band were not given; (6) “del” or “dup” was not used to describe the variant type; (7) the span of nucleotides was not provided; or (8) other errors were not consistent with the recommendations of ISCN and HGVS. However, errors belonging to the different items listed below were only penalized with a deduction of 1 point, since their impact with compliance of the nomenclature was minor (such as normal chromosomes were listed; an underscore was not used when indicating nucleotide range or a hyphen was used instead; the genome build was not placed in a square bracket or there was no space after the square bracket, or HGVS-like portions were not placed on a separate line, etc).

Overall, the compliant score was 7.89 ± 2.04 (mean score ± SD) out of 10 points. Only Hospital 16 received 10 points indicating they used a nomenclature that was fully ISCN 2016 and HGVS-compliant without any errors/inconsistencies assessed in this study. In contrast, only Hospital 3 had a score of 0 because they only provided a simple nomenclature of the affected band of the chromosome and the type of the variant without following the ISCN and HGVS nomenclature format. For the other participating hospitals with scores ranging from 6 to 9, they generally followed the nomenclature format; however, there were elements missing or inappropriately used. Among the hospitals submitting CNV nomenclatures in our survey, 2/19 (10.52%) did not include the symbol of sequencing-based technology, 1/19 (5.26%) did not mention the genome build, 10/19 (52.63%) did not provide the prefix of reference sequences “g.”, 2/19 (10.52%) did not state “del” or “dup” to describe the variant type, and 17/19 (89.47%) contained other errors mentioned previously. All participating hospitals described the affected chromosome as well as the affected chromosome arm and band in their nomenclatures. For the genome build presented in the nomenclature, although “GRCh37” was given as a reference in the questionnaire, more than half of the participants changed it to or added “hg19” in their nomenclature format.

## 4. Discussion

In 2019, a joint Chinese expert group’s consensus was published recommending low-pass WGS for clinical application [11]. However, neither international nor national guidelines contain detailed recommendations particularly for utilizing low-pass WGS in the application of CNV detection for both wet-lab and dry-lab procedures. In terms of the variant nomenclature in a clinical report, the knowledge of how well the existing guidelines were correctly implemented in China is still limited. In addition, the volume of clinical molecular genetic diagnostic testing in China was the highest in the world, therefore, conducting this survey to identify issues in clinical routine practice is of the utmost importance.

### 4.1. Low-Pass WGS-Based CNV Analysis Is in Great Demand in China

Our survey results revealed that low-pass WGS-based CNV analysis was applied as a diagnostic genetic test for high-risk pregnancies, spontaneous abortions, couples with an adverse pregnancy history, and children with congenital birth defects, which affect a significant portion of the population of China [20,21,22,23]. Spontaneous abortion accounted for the largest proportion of the cases referred for low-pass WGS-based CNV analysis. The underlying reason might be related to a longer turn-around-time allowed when compared with cases from prenatal diagnosis, and small CNVs underappreciated by low-pass CNVs might not be the causative factor for miscarriage. In addition, a large sample size might be another reason. Among all clinically recognized pregnancies, 6–15% ended in spontaneous abortion, predominantly due to chromosomal abnormalities [21], and over 1% were assisted reproductive couples who were more anxious about the etiology [22]. Couples with an adverse pregnancy history ranked as the second-largest referral group, which was expected to increase after the national three-child policy was released by the Chinese government in 2021. Although this group of patients would be eager for a diagnosis, advanced parental age may be the main contributor to the increasing incidence of pregnancy complications, which might not be directly reflected in their CNV analysis results from the peripheral blood. [23] In addition, if both partners of the couple were clinically asymptomatic, testing for balanced translocation or carrier status for autosomal recessive/X-linked conditions may be considered. For high-risk pregnancies and children with congenital birth defects, cases were referred for low-pass WGS-based CNV analysis due to highly suspected genetic causes of birth defects either prenatally or postnatally, which are estimated to be over 900,000 cases occurring in China annually [20]. Although CMA is widely used as the first tier of testing for clinically significant CNVs for the above indications across the globe [4,5], low-pass WGS is increasingly utilized in Chinese laboratories owing to its lower costs and manpower for both wet-lab and data-analysis procedures, and its high throughput with a shortened turnaround time [10], which are more favorable for application in China considering the large number of sample volumes for each indication mentioned above. Therefore, the demand for low-pass WGS-based CNV analysis will likely grow. However, according to our survey results, only 13 out of 21 participants applied low-pass WGS-based CNV analysis in prenatal diagnosis for high-risk pregnancies. The data also suggested that additional training programs in prenatal diagnosis techniques and qualification examinations might facilitate more hospitals to improve their service quality in providing services to meet the enormous demand for prenatal diagnosis testing in China. GS-based CNV analysis may be underutilized in pediatric cases with congenital birth defects because only one participant provided services relevant to pediatrics. However, there are several studies suggesting applying rapid high read-depth WGS as the first tier of testing for newborns in intensive care units (ICU) with suspected genetic diseases, and it is believed that it can lead to a timely and high-yield diagnostic and a reduction in total costs [24,25]. Further studies focusing on the comparison among different diagnostic strategies of Chinese hospitals are needed.

### 4.2. Various Patterns of Low-Pass WGS-Based CNV Analysis Bring Challenges to Quality Assessment

In China, the National Health Commission (NHC) is in charge of the certification of clinical laboratories. Initially, over 100 hospitals were accredited by the NHC in the first batch to conduct molecular testing using NGS technology in 2014 and 17 of our participants were included. The National Centre for Clinical Laboratory (NCCL) is responsible for the external quality assessment (EQA) for nationwide clinical laboratories and healthcare organizations. However, currently, there are no EQA programs for low-pass WGS-based CNV analysis testing. Possible reasons are a lack of standardization and the complex processes involved in low-pass WGS-based CNV analysis, which involves expensive costs associated with manpower from different disciplines, the use of reagents and equipment, and the bioinformatics pipeline and other computing resources. Unlike other straightforward testing, clinical laboratories may use several different approaches when conducting low-pass WGS-based CNV analysis [26]: (Pattern I) conduct the entirety of the testing within their laboratory; (Pattern II) outsource the wet-bench work to save money and then develop the bioinformatics pipeline and the interpretation workflow in-house; (Pattern III) process the wet-bench steps but outsource the data analysis and interpretation procedures to commercial companies due to a lack of specialist; (Pattern IV) outsource the whole procedure and only review the data or drafted reports returned to them, then sign and issue the reports. In our study, 57% reported Pattern I, 15% of participants adopted Pattern II, 28.6% used Pattern IV; however, none employed Pattern III. In this environment, it is a great challenge for quality assessment programs since wet- and dry-bench components may be performed by different organizations at different physical addresses. The increasing risk of sample mix ups or mislabeling, inadequate quality control checkpoints, inappropriate data interchange procedures, or other unknown factors should be considered [27]. Interestingly, in some situations such as Hospital 4, the purpose of using a high read depth was to find the structural rearrangements and absence of heterozygosity (AOH) besides CNV for couples with an adverse pregnancy history and children with congenital birth defects, whereas the total volume from this group was too low to run the wet bench internally.

### 4.3. Challenges Regarding the Establishment of Standard Criteria for Low-Pass WGS-Based CNV Analysis

In our study, the most commonly reported detection limit of CNV length was 100 kb, or even as precise as 50 kb, which detected most clinically pathogenic CNVs described in former cohort studies [13,14]. However, the definition of the detection limits for different indications based on larger cohort studies mapping the characteristics of clinically significant CNVs is still necessary as a reference for future quality assessment programs. As for the sequencing method, more hospitals utilized single-end sequencing, likely due to the increase in costs and sequencing time required by paired-end sequencing. Furthermore, single-end sequencing can already efficiently detect most CNVs unless detection for complex structural variants was needed. In this study, Hospitals 4 and 16 reported a more flexible process utilizing two sequencing methods for different clinical indications requiring different detection scopes, which might be a valuable approach for the establishment of future standards. Moreover, the results of our survey revealed that the average depth of coverage (determined by sequencing read length and read counts) is also varied even for the same detection limit, which also makes it challenging for cross-laboratory referencing or comparisons. The sufficient average depth of coverage to achieve an appropriate resolution also needs to be explicitly stipulated. As for more detailed considerations about the sequencing parameters and checkpoints to be included in future standard criteria, manufacturers of in vitro diagnostic (IVD) medical devices, such as MGI, Illumina, and Thermo Fisher Scientific, could provide valuable advice as technical consultants [28].

### 4.4. Challenges Regarding Implementation of Existing Criteria for CNV Nomenclature

In our study, we designed a scoring rubric based on the recommendations given by ISCN (2016) and HGVS to assess the compliance level of each description submitted. Our results showed that the mean score of participants was 7.79 (95% CI, 6.78–8.80), which represented an overall acceptable implementation of the existing gold standard criteria. However, the most common error was the lack of prefix of reference sequences followed by an absence of sequencing technology, variant type, or genome build. These errors resulted in a lack of specificity in the description of a variant and could result in other healthcare providers performing family validation for an incorrect variant, inappropriate interpretation of the clinical significance for the variant, or even providing misleading clinical guidance to patients. Therefore, it is critical to follow the format provided by professional committees. It is worth noting that Hospitals 5, 6, and 7 were using the same platform for low-pass WGS-based CNV analysis and shared the same format and errors, which may indicate that they referenced inappropriate nomenclatures provided by the same software. Therefore, it is important to manually check the automatically generated variant nomenclature in clinical practice. As for the official name for the human genome reference assembly, the Genome Reference Consortium Human Build 37 (abbreviated as GRCh37) is recommended, although it is referred to as hg19 in the UCSC Genome Browser; however, hg19 is not the official assembly’s name or abbreviation.

Moreover, the updated edition of the International System for Human Cytogenetic Nomenclature (ISCN 2020), has been published following a thorough revision of the 2016 issue after we conducted this study and it was suggested that all laboratories follow the new 2020 version of criteria starting from April 2020. Therefore, additional key points are to be added to these scoring criteria: the specific genomic reference sequence should be mentioned at the beginning of the HGVS portion; the affected chromosome only needs to be mentioned in the ISCN portion without duplication in the HGVS portion;, and a recommendation to use GRCh38 rather than GRCh37 in the new edition, etc. [29].

## 5. Conclusions

In summary, our results indicated that the low-pass WGS-based CNV analysis laboratory service is in great demand in China though it is still in the early stages of its development and requires standardization. From a quality-control perspective, challenges remain regarding the establishment of standard criteria for low-pass WGS-based CNV analysis and data-reporting formats. The low-pass WGS-based method is becoming a common diagnostic approach, transforming the possibilities for genetic diagnoses for patients in China.

## Figures and Tables

**Table 1:** Characteristics of the 24 participating hospitals.

Participant Code	Hospital Code	Hospital Location	Low-Pass WGS-Based CNV Analysis	Department of Participant	Low-Pass WGS-Related Services			
					General Molecular Genetic Testing	Assisted Reproduction	Prenatal Diagnosis	Pediatrics
P1	Hospital 1	Hefei, Anhui	Y	Reproductive Medicine Center	Y	Y	N	N
P2	Hospital 2	Beijing, Beijing	Y	Obstetrics Centre	Y	N	Y	N
P3	Hospital 3	Shanghai, Shanghai	Y	Pediatrics Research Institute	N	N	N	Y
P4	Hospital 4	Shanghai, Shanghai	Y	Prenatal Diagnosis Center	Y	N	Y	N
P5	Hospital 5	Guangzhou, Guangdong	Y	Laboratory of the Obstetrics and Gynecology Institute	Y	N	Y	N
P6	Hospital 6	Guiyang, Guizhou	Y	Reproductive Medicine Centre	N	Y	N	N
P7	Hospital 7	Shijiazhuang, Hebei	Y	Reproductive Medicine Centre	N	Y	N	N
P8	Hospital 8	Changsha, Hunan	Y	Genetics Centre	Y	N	N	N
P9	Hospital 9	Nanchang, Jiangxi	Y	Prenatal Diagnosis Center	Y	N	Y	N
P10	Hospital 10	Xi’an, Shaanxi	Y	Obstetrics and Gynecology Centre	N	Y	N	N
P11	Hospital 11	Jinan, Shandong	Y	Department of Reproductive Genetics	Y	N	N	N
P12	Hospital 12	Taiyuan, Shanxi	Y	Reproductive Medicine Center	N	Y	N	N
P13	Hospital 13	Shanghai, Shanghai	N	Shanghai Key Laboratory of Maternal Fetal Medicine, Department of Fetal Medicine & Prenatal Diagnosis Center	Y	N	Y	N
P14	Hospital 14	Chengdu, Sichuan	N	Reproductive Medicine Centre	N	Y	N	N
P15	Hospital 15	Suzhou, Jiangsu	Y	Centre of Reproduction and Genetics	Y	Y	Y	N
P16	Hospital 16	Shatin, Hong Kong SAR	Y	Prenatal Genetic Diagnosis Centre, Department of Obstetrics & Gynecology	Y	N	N	N
P17	Hospital 17	Urumqi, Xinjiang	Y	Prenatal Diagnosis Center	Y	Y	Y	N
P18	Hospital 18	Kunming, Yunnan	Y	Department of Genetics Medicine	Y	N	Y	N
P19	Hospital 19	Hangzhou, Zhejiang	Y	Reproductive Genetics Centre	Y	N	N	N
P20	Hospital 20	Changsha, Hunan	Y	Genetics Centre	Y	N	N	N
P21	Hospital 21	Shenyang, Liaoning	Y	Reproductive Medicine Centre	N	Y	N	N
P22	Hospital 22	Fuzhou, Fujian	Y	Centre of Reproductive Medicine	N	Y	N	N
P23	Hospital 23	Wuhan, Hubei	N	Reproductive Medicine Centre	Y	Y	Y	N
P24	Hospital 24	Zhengzhou, Henan	Y	Reproductive Medicine Centre	N	Y	N	N
-	-	-	21/24 (87.5%)	-	15/24 (62.5%)	12/24 (50.0%)	9/24 (37.5%)	1/24 (4.2%)

Low-pass WGS-based CNV analysis: next-generation sequencing-based copy number variant analysis; Y: the department provided the service; N: the department did not provide the service.

**Table 2:** Sequencing platforms and manufacturers utilized by 21 Chinese tertiary hospitals in this survey.

Manufacturer	Platform	No. of Hospital
MGI	MGISEQ-2000	3
	BGISEQ-500	1
Illumina	NovaSeq 6000	2
	NextSeq 500	2
	Annoroad NextSeq 550AR	1
	Berry Genomics NextSeq CN500	4
	MiSeq/MiSeqDx	2
	HiSeq 2000	1
Thermo Fisher Scientific	Ion Proton	6

**Table 3:** Overview of variant nomenclatures submitted by participating hospitals.

Hospital Code	Manufacture	Platform	DiGeorge Syndrome (DGS) seq[GRCh37]del(22)(q11.21) chr22:g.19009792_21452445del	Charcot-Marie-Tooth Disease Type 1 (CMT1) seq[GRCh37]dup(17)(p12) chr17:g.14097915_15470903dup	Score Points										
					+a	+b	−c	−d	−e	−f	−g	−h	−i	−ji	Sum
Hospital 1	Thermo Fisher	Ion proton	seq[GCRh37]del(22)(q11.2)#chr22:g.19009792-21452445del	seq[GCRh37]dup(17)(p12)#chr17:g.14097915-15470903dup	5	5	0	0	0	0	0	0	0	1	9
*Hospital 2*	Illumina	NovaSeq 6000	seq[GRCh37] 22q11.2(19009792_21452445)X1	seq[GRCh37] 17p12(14097915_15470903)X3	5	5	0	0	1	0	0	1	0	1	7
Hospital 3	Illumina	NovaSeq	There may be 22q11 microdeletion syndrome	There may be duplication on chromosome 17p12	0	0	-	-	-	-	-	-	-	-	0
Hospital 4	Illumina	Berry Genomics NextSeq CN500, HiSeq 2000	seq[hg19]del(22)(q11.2) chr22:g.19009792_21452445del	seq[hg19]dup(17)(p12) chr17:g.14097915_15470903dup	5	5	0	0	0	0	0	0	0	1	9
Hospital 5	Thermo Fisher	Ion proton	del(22)(q11.2).seq[GRCh37](19009792-21452445)×1	dup(17)(p12).seq[GRCh37](14097915-15470903)×3	5	5	0	0	1	0	0	0	0	1	8
*Hospital 6*	Thermo Fisher	Ion proton	del(22)(q11.2).seq[GRCh37/hg19](19009792-21452445)×1	dup(17)(p12).seq[GRCh37/hg19](14097915-15470903)×3	5	5	0	0	1	0	0	0	0	1	8
Hospital 7	Thermo Fisher	Ion proton	del(22)(q11.2).seq[GRCh37/hg19](19009792-21452445)X1	dup(17)(p12).seq[GRCh37/hg19](14097915-15470903)X3	5	5	0	0	1	0	0	0	0	1	8
Hospital 8	Illumina	Berry Genomics NextSeq CN500	seq[hg19]del(22)(q11.2)#chr22:g.19009792_21452445del	seq[hg19]dup(17)(p12)#chr17:g.14097915_15470903dup	5	5	0	0	0	0	0	0	0	1	9
*Hospital 9*	BGI	MGISEQ-2000	seq[GRCh37] del(22)(q11.2)chr22:g.19009792_21452445 del	seq[GRCh37] dup(17)(p12)chr17:g.14097915_15470903 dup	5	5	0	0	0	0	0	0	0	1	9
Hospital 10	Illumina	MiSeqDx	del(22)(q11.2).(19009792-21452445)X1	dup(17)(p12).(14097915-15470903)X3	5	5	1	1	1	0	0	0	0	1	6
Hospital 11	Illumina	NextSeq 500	seq[hg19] 22q11.2(19009792-21452445 )x1 CNV type: heterozygous deletion length: 2.3 Mb classification: pathogenic	seq[hg19] 17p12(14097915-15470903)x3 CNV type: duplication length: 1.3Mb classification:pathogenic	5	5	0	0	1	0	0	0	0	1	8
*Hospital 12*	BGI	MGISEQ-2000	46,XN,del(22q11.2).seq[GRCh37/hg19](19009792-21452445)x1	46,XN,dup(17p12).seq[GRCh37/hg19](14097915-15470903)x3	5	5	0	0	1	0	0	0	0	1	8
Hospital 15	Illumina	MiSeq	seq[GRCh37]del(22)(q11.2)(19009792-21452445)	seq[GRCh37]dup(17)(p12)(14097915-15470903)	5	5	0	0	1	0	0	0	0	1	8
Hospital 16	BGI	MGISEQ-2000	seq[GRCh37] del(22)(q11.21) mat/pat/dn chr22:g.19009792_21452445del	seq[GRCh37] dup(17)(p12) mat/pat/dn chr17:g.14097915_15470903dup	5	5	0	0	0	0	0	0	0	0	10
Hospital 17	Thermo Fisher	Ion proton	del(22)(q11.2).seq[GRCh37/hg19](19009792-21452445)X1	dup(17)(p12).seq[GRCh37/hg19](14097915-15470903)X3	5	5	0	0	1	0	0	0	0	1	8
Hospital 18	Illumina	NextSeq 550AR	DiGeorge syndrome #band: 22q11.2 #Genomic coordinate (GRCh37) 22:g.19009792-21452445 #type: heterozygous deletion	CMT syndrome type 1 #band: 17p12 #Genomic coordinate (GRCh37) 17:g.1409795-15470903 #type: duplication	5	5	1	0	0	0	0	0	0	1	8
Hospital 20	Thermo Fisher	Ion proton	seq[hg19] 22q11.21(18620001_21820000)X1	seq[hg19] 17p12(14097915_15470903)X3	5	5	0	0	1	0	0	1	0	1	7
Hospital 21	Illumina	NextSeq 500	seq[hg19]del(22)(q11.2) chr22:g.19009792_21452445del	seq[hg19]dup(17)(p12) chr17:g.14097915_15470903dup	5	5	0	0	0	0	0	0	0	1	9
*Hospital 22*	Illumina	Berry Genomics NextSeq CN500	DGS seq(hg19)del(22)(q11.2) chr22:g.19009792_21452445dup	CMT seq(hg19)dup(17)(p12) chr17:g.14097915_15470903dup	5	5	0	0	0	0	0	0	0	1	9
19 hospitals	3 manufacturers	11 Platforms	12 types of nomenclature	12 types of nomenclature	1#	1#	2&	1&	10&	0&	0&	2&	0&	17&	7.79 (95% CI, 6.78–8.80) *

Hospital Code: Shaded text indicates that the analytic wet-bench process of low-pass WGS-based CNV analysis was outsourced; Italicized text indicates that the bioinformatics analysis process of low-pass WGS-based CNV analysis was outsourced. Key Points: a. ISCN-like nomenclature format for chromosomal aberrations used, +5 points; b. HGVS-like nomenclature format for nucleotide variant used, +5 points; c. sequencing-based technology not mentioned, −1 point; d. genome build not mentioned, −1 point; e. prefix reference sequences not mentioned, −1 point; f. affected chromosome not listed for ISCN-like or HGVS-like portions, −1 point; g. affected arm and band not mentioned, −1 point; h. del/dup not used to describe the variant type, −1 point; i. span of nucleotides not mentioned, −1 point; j. other errors (normal chromosomes listed, an underscore was not used when indicating nucleotide range or a hyphen was used instead, genome build not in a square bracket or no space after it, HGVS-like portion not on separate line, etc., −1 point); X or × both indicating the number of times the variant was observed, initial signs in the nomenclature submitted by participants were retained to show the real situation; **#** Number of hospitals not following the nomenclature format; & Number of hospitals with these types of errors; * Mean (95% CI).

## Data Availability

The original contributions presented in the study are included in the article, further inquiries can be directed to the corresponding authors.

## Appendix A

The Chinese Genomic Structural Variants Consortium Authors and Affiliations List

Kwong Wai Choy ^1^, Ye Cao ^1^, Zirui Dong ^1^, Baosheng Zhu ^2^, Jichun Tan ^3^, Yichun Guan ^4^, Yulin Jiang ^5^, Hua Wang ^6^, Guangxiu Lu ^7^, Ge Lin ^7^, Yueqiu Tan ^7^, Qun Lv ^8^, Beihong Zheng ^9^, Qing Li ^10^, Shuyun Zhao ^11^, Minyue Dong ^12^, Yunxia Cao ^13^, Junhao Yan ^14^, Yanqiu Liu ^15^, Wenhao Zhou ^16^, Xiaoxi Sun ^17^, Caixia Lei ^17^, Feng Zhang ^17^, Luming Sun ^18^, Guimin Hao ^19^, Hong Li ^20^, Xueqing Wu ^21^, Weidong Huang ^22^, Jing Yang ^23^, Xiaohong Wang ^24^.

Affiliations:

1 Prenatal Genetic Diagnosis Centre, Department of Obstetrics & Gynecology, Prince of Wales Hospital, The Chinese University of Hong Kong, Hong Kong, China
2 Department of Genetics Medicine, First People’s Hospital of Yunnan Province, Kunming 650021, China
3 Reproductive Medicine Centre, Shengjing Hospital of China Medical University, Shenyang 110055, China
4 Reproductive Medicine Centre, Third Affiliated Hospital of Zhengzhou University, Zhengzhou 450052, China
5 Obstetrics Centre, Peking Union Medical College Hospital, Beijing 100006, China
6 Genetics Centre, Hunan Provincial Maternal and Child Health Care Hospital, Changsha 410008, China
7 Genetics Centre, Reproductive & Genetic Hospital of CITIC-Xiangya, Changsha 410008, China
8 Reproductive Medicine Centre, Sichuan Academy of Medical Sciences & Sichuan Provincial People’s Hospital, Chengdu 610032, China
9 Centre of Reproductive Medicine, Fujian Maternity and Child Health Hospital, Affiliated Hospital of Fujian Medical University, Fuzhou 350004, China
10 Laboratory of the Obstetrics and Gynecology Institute, The Third Affiliated Hospital of Guangzhou Medical University, Guangzhou 510150, China
11 Reproductive Medicine Centre, The Affiliated Hospital of Guizhou Medical University, Guiyang 550031, China
12 Reproductive Genetics Centre, Women’s Hospital School of Medicine Zhejiang University, Hangzhou 310006, China
13 Reproductive Medicine Center, The First Affiliated Hospital of Anhui Medical University, Hefei 230022, China
14 Department of Reproductive Genetics, Hospital for Reproductive Medicine Affiliated to Shandong University, Jinan 250021, China
15 Prenatal Diagnosis Center, Jiangxi Maternal and Child Health Hospital, Nanchang 330006, China
16 Pediatrics Research Institute, The Children’s Hospital of Fudan University, Shanghai 201102, China
17 Prenatal Diagnosis Center, Shanghai JIAI Genetics & IVF Institute, Obstetrics & Gynecology Hospital of Fudan University, Shanghai 200082, China
18 Shanghai Key Laboratory of Maternal Fetal Medicine, Department of Fetal Medicine & Prenatal Diagnosis Center, Shanghai First Maternity and Infant Hospital, School of Medicine, Tongji University, Shanghai 200125, China
19 Reproductive Medicine Centre, The Second Hospital of Hebei Medical University, Shijiazhuang 050004, China
20 Centre of Reproduction and Genetics, Suzhou Municipal Hospital, Suzhou 215005, China
21 Reproductive Medicine Center, Children’s Hospital of Shanxi (Women Health Center of Shanxi), Taiyuan 030083, China
22 Prenatal Diagnosis Center, Xinjiang Jiaying Hospital, Urumqi 830041, China
23 Reproductive Medicine Centre, Renmin Hospital of Wuhan University, Wuhan 430064, China
24 Obstetrics and Gynecology Centre, Tangdu Hospital, Xi’an 710024, China
